# Clinical and anatomical characteristics of basal temporal seizures: A systematic review

**DOI:** 10.1002/epd2.70020

**Published:** 2025-04-04

**Authors:** Fabrice Bartolomei, Francesca Pizzo, Stanislas Lagarde

**Affiliations:** ^1^ APHM, Timone Hospital, Epileptology and Cerebral Rhythmology Marseille France; ^2^ Aix Marseille Univ, INSERM, INS, Inst Neurosci Syst Marseille France

**Keywords:** basal temporal, drug‐resistant epilepsy, epilepsy surgery, focal epilepsy, SEEG, semiology, temporal lobe epilepsie

## Abstract

This review aimed to characterize the clinical semiology and anatomical correlates of seizures originating in the basal temporal region, an underrecognized epilepsy subtype, and to identify features that distinguish it from other forms of temporal lobe epilepsies (TLE). We performed a systematic review of the literature following PRISMA guidelines. The search included terms related to the basal temporal region (e.g., fusiform gyrus and rhinal cortex) and epilepsy, encompassing clinical, anatomical, and neurophysiological studies. Studies with video‐EEG monitoring, SEEG evaluations, and surgical outcomes were prioritized. Semiological features, imaging findings, and post‐surgical outcomes were extracted and analyzed. Fifteen studies encompassing 83 patients were analyzed. Most cases involved MRI‐detectable lesions. Findings revealed that basal temporal seizures frequently present with language disturbances, motor phenomena, and less pronounced emotional and sensory signs compared to other TLE forms. SEEG identified epileptogenic zones predominantly in the fusiform gyrus, rhinal cortices, and parahippocampal region. Post‐surgical outcomes revealed 57% of patients achieving Engel Class I results after 1 year, highlighting the potential benefits of accurate diagnosis and intervention. However, diagnostic challenges persist due to overlapping with other TLE subtypes, emphasizing the importance of advanced imaging and SEEG. Further studies are needed to refine diagnostic criteria and improve understanding of the functional implications of basal temporal epilepsies.

**Key points**

- Basal temporal seizures are a rare and under recognized subtype of temporal lobe epilepsy.
- Seizures originating in the basal temporal region are commonly associated with language disturbances (particularly aphasia and jargonaphasia), motor phenomena, and relatively fewer emotional or visceral auras than mesial temporal lobe epilepsy.
- The epileptogenic zone is most often located in the fusiform gyrus, rhinal cortices, and parahippocampal region, as demonstrated by SEEG recordings and lesion localization.
- SEEG is essential for accurately identifying basal temporal epilepsy, as scalp EEG and standard imaging techniques may fail to capture the activity confined to deep basal structures.

## 1 INTRODUCTION

Basal temporal seizures represent a rare and often under‐recognized subtype of epilepsy, which has not been explicitly classified in major epilepsy taxonomies, such as the 1989 International League Against Epilepsy (ILAE) classification^1^ or even more recent efforts.^2^ Historically, mentions of basal temporal seizures have been limited to isolated case reports or case series,^3^, ^4^ and they have often been overshadowed by more well‐known temporal lobe epilepsy (TLE) syndromes, such as mesial temporal lobe epilepsy (MTLE). However, recent studies^5^, ^6^, ^7^, ^8^ suggest a growing interest in understanding the unique characteristics of basal temporal seizures, likely due to advances in neuroimaging and intracranial EEG monitoring.

One reason for the under‐representation of basal temporal seizures in epilepsy literature could be the anatomical complexity of the basal temporal region. This region includes the fusiform gyrus, bordered by two prominent sulci (collateral sulcus medially and occipitotemporal sulcus laterally), which separate it from the parahippocampal gyrus medially and the inferior temporal cortex laterally.^9^, ^10^, ^11^ The anterior part is in contiguity with the temporal polar region, while the posterior limit is in close proximity to the inferior occipital sulcus. As a result, basal temporal epilepsies are challenging to differentiate from other forms of TLE, such as temporo‐polar or mesial lateral TLE,^12^, ^13^, ^14^ mesial TLE and lateral TLE, as well as from occipito‐temporal epilepsies.^15^, ^16^ Additionally, the historical positioning of stereoelectroencephalography (SEEG) electrodes, often placed above the level of the basal temporal region, may fail to adequately capture activity from this region, further complicating diagnosis. Similarly, ECoG cannot effectively explore the collateral and occipitotemporal sulci, potentially missing restricted epileptogenic zones located within these regions.

Despite these challenges, the functional significance of the temporo‐basal region is substantial. The fusiform gyrus, for instance, is known to be involved in complex cognitive processes such as visual perception,^17^ semantic processing, including reading^18^ and language.^10^, ^19^, ^20^, ^21^, ^22^ Particularly, the basal temporal area in the dominant hemisphere has been called the basal temporal‐language area (BTLA).^20^, ^21^ The resection of the BTLA region (1–9 cm apart from the tip of the temporal pole, with inter‐patient variability) is associated with a risk of naming decline after anterior temporal lobectomy.^23^ However, a recent study on patients undergoing SEEG with longer follow‐up showed a partial recovery of language deficit with time after BTLA resection.^24^ The basal temporal cortex is a highly integrative area linked to semantic, emotional, and memory networks.^25^ For instance, the fusiform gyrus also has extensive connections with other brain regions, including the occipital cortex via the inferior longitudinal fasciculus (ILF) and the prefrontal cortex via the inferior frontal‐occipital fasciculus (IFOF).^26^, ^27^ This intricate network of short‐ and long‐range connections underscores the region's importance in cognitive function, making seizures originating from this area particularly disruptive to language and memory.

Basal temporal epilepsies can thus be defined as epilepsies with maximum epileptogenicity in the fusiform gyrus, the collateral, and occipital‐temporal sulci, and which may spill over into the parahippocampal gyrus medially.^7^

Given the increasing recognition of the basal temporal region's functional and clinical significance, more detailed studies that focus specifically on this form of epilepsy are needed. In this perspective, we performed a systematic review of the ictal semiology of the basal temporal region in focal epilepsy to summarize the state‐of‐the‐art anatomy correlations in the field and help guide the interpretation of ictal semiology within the framework of pre‐surgical evaluation.

## 2 MATERIALS AND METHODS

### 2.1 Search strategy and eligibility criteria

We conducted a systematic review of the published evidence and report its results according to the Preferred Reporting Items for Systematic Review and Meta‐Analysis (PRISMA)statement.^28^ The search was performed using a combination of key terms that reflect the anatomical focus and seizure characteristics, including: “basal AND temporal/parahippocampal OR parahippocampus/fusiform/rhinal OR perirhinal OR entorhinal/collateral/occipitotemporal sulcus” in conjunction with “epilep* OR seizure,” and further combined with terms related to seizure diagnosis and management, such as “(surgery* OR EEG)/(video)/(semiology).” The databases used for the search included PubMed, Scopus, and Web of Science, covering publications up to December 2023.

### 2.2 Inclusion and exclusion criteria

We primarily selected studies published as papers in peer‐reviewed journals with an abstract available, limited to English, French, German, Spanish, or Italian. Given the rarity of basal temporal seizures, no strict limits were set for the number of patients in each study. Studies were selected based on their descriptions of seizure semiology, stimulation outcomes, post‐surgical results, electrical source imaging, and anatomical findings. Specific inclusion criteria also encompassed the presence of video‐EEG monitoring and a focus on basal temporal epilepsies. Studies limited to the stimulation of this region were not included. Two independent reviewers screened titles, abstracts, and full‐text articles to determine eligibility. A third reviewer resolved disagreements at the full‐text screening phase and the data abstraction phase.

### 2.3 Data extraction

For each identified publication, we extracted all patients with informative data regarding the topic of this review, with special attention to data on anatomo‐clinical correlations.

Although not easily applicable to the combination of case reports, small case series, and literature‐based identification of single patients, nor to the evaluation of many‐tiered anatomy–clinical associations, we performed a QUADAS2‐guided assessment regarding both patient enrollment and the assessment of symptoms.^29^

### 2.4 Reliability of the reference standard

For each identified case, we assessed our level of confidence in the reported epileptogenic zone (EZ) according to a recently developed method.^30^ Based on the findings from MRI, intracerebral EEG (iEEG) and post‐operative outcome, four levels of evidence were distinguished:

1. “very high” confidence in the reported EZ for patients with Engel class IA after at least 1 year of post‐operative follow‐up;
2. “high” confidence in the reported EZ for patients with either: (i) a well delineated focal lesion suspected to represent at least part of the EZ (according to the authors of the publication), or (ii) a well delineated EZ according to all available iEEG data (according to the authors of the publication), or (iii) an Engel class I (but not specified IA) after at least 1 year of post‐operative follow‐up;
3. “moderate” confidence in the reported EZ for patients with MRI signs of hippocampal sclerosis or atrophy suspected to be at least part of the EZ;
4. “low” confidence in the reported EZ for patients whose MRI would be normal or show multilobar, multifocal, or poorly delineated lesion, or with a poorly delineated EZ according to all available iEEG data (according to the authors of the publication), or an Engel class II‐IV post‐operative outcome provided the entire EZ has been entirely removed. Surgical failure in patients whose suspected EZ would not have been fully removed would not be considered for grading.

If several of the above items were available and provided different levels of confidence, that associated with the post‐operative outcome prevailed over the iEEG and MRI findings, while the iEEG conclusion would prevail over the MRI findings.

For the result of surgery, we considered only selective/tailored surgery for a high evidence grading.

### 2.5 Statistical analysis

Descriptive statistics were used to summarize patient demographics, imaging findings, and surgical outcomes. The frequency of various semiological features (e.g., motor symptoms and language disturbances) was calculated. Where possible, studies with larger patient cohorts were compared to assess variability in results, and key patterns in clinical presentation were identified across different studies.

## 3 RESULTS

### 3.1 Study selection

**FIGURE 1:**
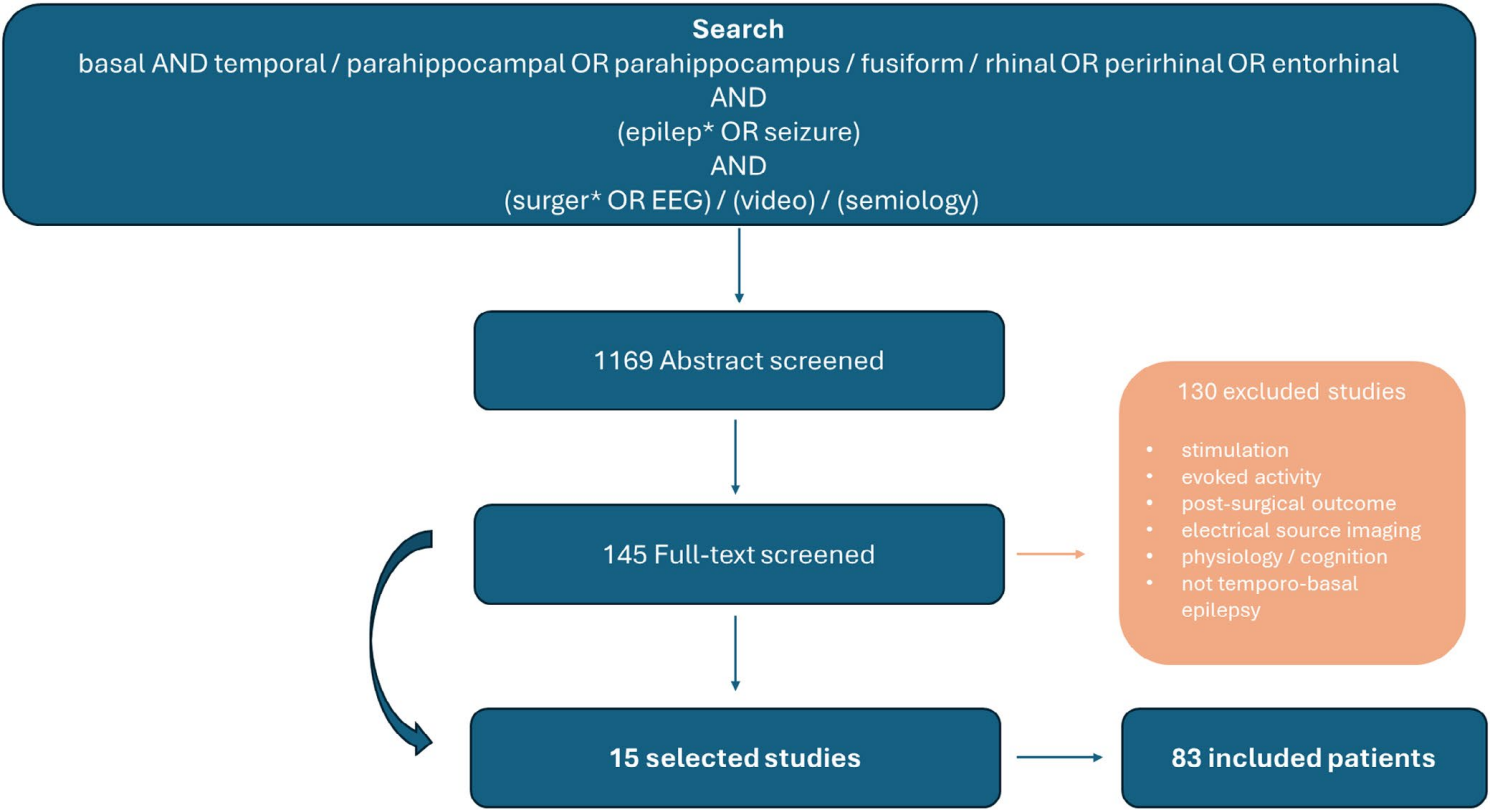
The PRISMA flow diagram illustrates the systematic review process for studies on basal temporal epilepsy. The initial search identified 1169 abstracts screened using key terms related to the basal temporal region and epilepsy. After abstract screening, 145 full‐text articles were assessed for eligibility. Of these, 130 studies were excluded for reasons such as a lack of focus on temporobasal epilepsy, unrelated physiological or cognitive outcomes, or absence of relevant data like SEEG findings or surgical results. Additional references discussed in the articles were included post‐screening to enhance the review. The final selection included 15 studies representing 83 patients.

From an initial pool of 1169 abstracts, 145 full‐text articles were reviewed. The final selection included 15 studies, representing a total of 83 patients with confirmed basal temporal epilepsy. Seventy‐four percent benefited from SEEG or intracranial exploration, and 83% from surgical intervention, resulting in 87% achieving Engel class I outcomes. The PRISMA flow diagram for the search is shown in Figure 1. Confidence in identifying the epileptogenic zone (EZ) varied, with 4 cases rated as low, 1 moderate, 1 high, and 10 as very high.

Tables 1 and 2 summarize the main data from the 15 studies.

**TABLE 1:** The table presents detailed information on individual study results.

# study	# patients	Adult (A) Children (P)	% MRI positive	% SEEG	% operated	% class I ≥ 1 year	Confidence in the EZ	Was a consecutive or random sample of patients enrolled?	Was a case‐control design used?	Did the study avoid inappropriate exclusions?	Selection Bias Risk	Was semiology interpreted blinded to other data?	Assessment Bias Risk
1	24	17 A / 7 P	83%	100%	50%	67%	Very high (7) high (17)	Unclear	No	Unclear	High	Unclear	High
2	14	NA	100%	0%	100%	100%	High (10) moderate (4)	Unclear	No	Unclear	High	Unclear	High
3	13	3A / 10 P	85%	100% (mix)	100%	69%	Very high (10) high (2) low (1)	Unclear	No	Unclear	High	Unclear	High
4	9	NA	100%	100% (Mix w. ECoG)	100%	NA	Moderate	Unclear	No	Unclear	High	Unclear	High
5	5	4A / 1P	100%	0%	60%	100%	Very high (3) Moderate(2)	No	No	Unclear	High	Unclear	High
6	5	5A	100%	100%	100%	80%	Very high (4) High (1)	Unclear	No	Unclear	High	Unclear	High
7	3	2A /1P	100%	67%	100%	100%	Very high	No	No	Unclear	High	Unclear	High
8	3	3P	67%	100%	67%	100%	Very high	No	No	Unclear	High	Unclear	High
9	1	1A	100%	100% (ECoG)	100%	100%	Very high	No	No	N/A	High	Unclear	High
10	1	1A	100%	100% (ECoG)	100%	100%	Very high	No	No	N/A	High	Unclear	High
11	1	1A	100%	100% (ECoG)	100%	100%	Very high	No	No	N/A	High	Unclear	High
12	1	1P	100%	100%	100%	100%	Very high	No	No	N/A	High	Unclear	High
13	1	1A	100%	0%	100%	100%	Vey high	No	No	N/A	High	Unclear	High
14	1	1A	100%	0%	100%	100%	Very high	No	No	N/A	High	Unclear	High
15	1	1A	100%	100%	100%	100%	Very high	No	No	N/A	High	Unclear	High

*Note*: This table summarizes the patient demographics, imaging findings, surgical outcomes, and confidence levels in identifying the epileptogenic zone (EZ) across 15 studies on basal temporal seizures. It includes data on the number of patients (adults A vs. Pediatric P), MRI positivity, the use of SEEG, surgical intervention, Engel Class I outcomes at one year, and the confidence in EZ identification (rated as very high, high, moderate, or low). Abbreviation: NA, not available.

**TABLE 2:** This table also reports the total number of patients, with a breakdown of those categorized by their confidence in the EZ.

	# patients	% Adults	% MRI positive	% SEEG	% operated	% class I ≥ 1 year	High or very high confidence in the EZ
Min.	1	0	67	0	50	66.7	low
Max.	24	100	100	100	100	100	very high
Median	3	55.7	100	83.3%	100	100	NA
Total (=number of patient)	83	34 (40%)	79 (95%)	64 (77%)	71 (88%)	60/69 (87%)	Very high: 37; high:30 moderate:15; low:1

*Note*: Aggregated data across the 15 studies of basal temporal seizures. It includes the median values for adult percentage, MRI positivity, SEEG utilization, surgical outcomes, Engel Class I results after one year, and confidence levels in the epileptogenic zone.

According to QUADAS‐2 assessment (Table 1), the overall risk of bias across the studies is high, mainly due to non‐random patient selection. Most studies are retrospective, with small sample sizes and no control groups, limiting generalizability. Overall, while some studies demonstrated robust methodologies with well‐documented SEEG findings and surgical follow‐up, others had notable methodological limitations.

### 3.2 Study characteristics

The 15 selected studies included 86 patients, ranging in age from 9.5 to 53 years. The majority of the patients (55%) were adults. Most patients (76%) had positive MRI findings, with lesions identified in the fusiform gyrus, parahippocampal gyrus, or adjacent temporal structures. Confidence in identifying the epileptogenic zone was very high in 10 studies, moderate in 1, and low in 4.

### 3.3 Semiology and clinical features

**FIGURE 2:**
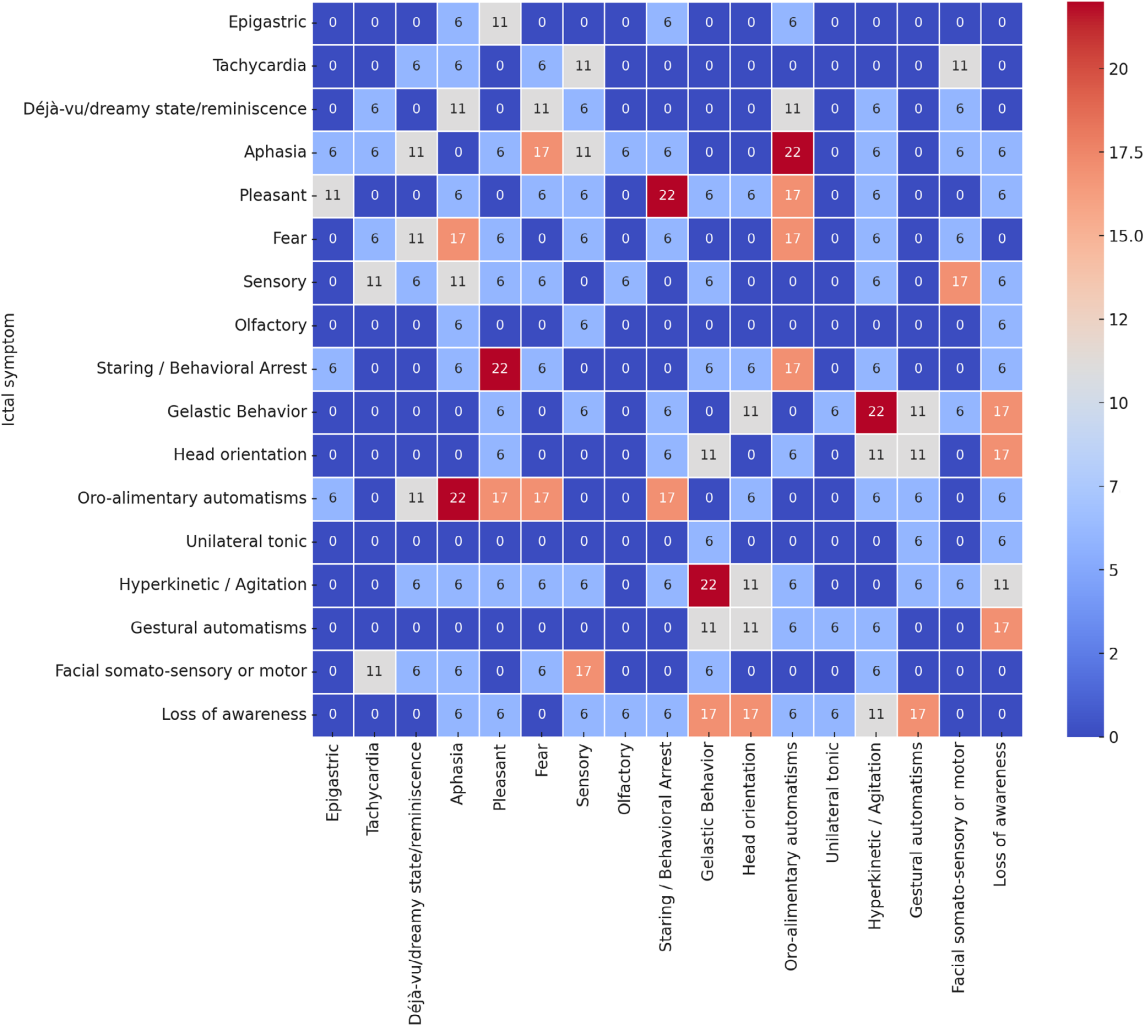
Co‐occurrence matrix of the main clinical signs and symptoms in 18 patients with basal temporal seizures detailed in some studies 18 patients.^4^, ^6^, ^8^, ^31^, ^32^, ^35^, ^36^, ^37^, ^38^, ^39^

The main semiological features are indicated in Table 3. Autonomic phenomena, such as rubefaction and tachycardia, were observed in 27% of cases, while classical features of mesial temporal lobe seizures, such as epigastric sensations, were rare (5%). Emotional subjective symptoms were infrequent (8%, including five cases of ictal pleasant sensation). Cognitive disturbances were reported in 41% of patients (aphasia (27%), déjà‐vu, and dreamy states (14%)), and sensory sensations in 27% (vertigo 8%, gustatory 7%, visual 5%, somatosensory 2%). The most common objective ictal signs included staring or behavioral arrest (42%), gestural automatisms (25%), head version or orientation (30%), unilateral tonic changes (25%), hyperkinetic behavior (13%), and facial somatomotor or sensory modifications (10%). Oro‐alimentary automatisms were the least frequent, occurring in just 7% of cases. Additionally, gelastic behavior has been reported in 6 cases of basal temporal seizures.^31^, ^32^ De la Vaissière et al.^33^ using SEEG recordings, also reported that lesions in the basal temporal cortex (three patients with DNET) could manifest with motor expressions such as bilateral spasms. Table 4 shows the analysis of 60 cases classified with high or very high confidence in the identification of the epileptogenic zone (EZ). The data from^34^ did not allow for the extraction of semiological features of only the patients with very high or high confidence in the epileptogenic zone, precluding its inclusion in this additional analysis. In addition, we analyzed the co‐occurrence of signs and symptoms from the detailed data obtained in 18 patients (small series and case reports).^4^, ^6^, ^8^, ^31^, ^32^, ^35^, ^36^, ^37^, ^38^, ^39^ Figure 2 shows a co‐occurrence matrix in these cases. The most frequent associations found were: oro‐alimentary automatisms and Aphasia (22%), hyperkinetic/agitation and gelastic behavior (22%), and staring/behavioral arrest & Pleasant (22%).

**TABLE 3:** This table outlines the frequency and type of ictal symptoms reported in basal temporal seizures.

Ictal symptom	% reported	*n*	More at onset or during propagation
Autonomic phenomena	27%	22	Onset
Nausea	4%	3	Onset
Epigastric	4%	3	Onset
Rubefaction	19%	16	Onset
Hyperventilation	2%	2	Onset
Tachycardia	4%	3	Onset
Déjà‐vu/dreamy state/reminiscence	14%	12	Onset
Aphasia	27%	22	Both
Emotional	8%	7	Onset
Pleasant	6%	5	Onset
Fear	1%	1	Onset
Sensory	27%	22	Onset/propagation?
Visual	5%	4	Onset
Gustatory	7%	6	Propagation?
Somato‐sensory	2%	2	Propagation?
Vertigo	8%	7	Onset
Olfactory	1%	1	Onset
Staring/Behavioral Arrest	42%	35	Propagation
Gelastic Behavior	7%	6	Propagation
Head orientation	30%	25	Propagation
Oro‐alimentary automatisms	7%	6	Propagation
Unilateral tonic	25%	21	Propagation
Hyperkinetic/Agitation	13%	11	Propagation
Gestural automatisms	25%	21	Propagation
Epileptic spasms	4%	3	Propagation
Facial somato‐sensory or motor	10%	8	Propagation

*Note*: It categorizes symptoms based on whether they are more common at the onset or during propagation of the seizure. Symptoms include autonomic phenomena, sensory disturbances, emotional experiences, and motor manifestations such as aphasia, gelastic behavior, and hyperkinetic movements.

**TABLE 4:** This table outlines the frequency and type of ictal symptoms reported in basal temporal seizures across 60 cases with very high or high levels of confidence in the epileptogenic zone (EZ).

Ictal symptom	reported	% (*n* = 60)
Autonomic phenomena	18	30%
Nausea	2	3%
Epigastric	4	7%
Rubefaction	7	12%
Hyperventilation	1	2%
Tachycardia	4	7%
Déjà‐vu/dreamy state/reminiscence	4	7%
Aphasia	21	35%
Emotional	9	15%
Pleasant	6	10%
Fear	4	7%
Sensory	15	25%
Visual	2	3%
Gustatory	4	7%
Somato‐sensory	1	2%
Vertigo	4	7%
Olfactory	1	2%
Staring / Behavioral Arrest	17	28%
Gelastic Behavior	5	8%
Head orientation	15	25%
Oro‐alimentary automatisms	23	38%
Unilateral tonic	9	15%
Hyperkinetic / Agitation	21	35%
Gestural automatisms	26	43%
Epileptic spasms	3	5%
Facial somato‐sensory or motor	3	5%
Loss of awareness	27	45%

Finally, only a few studies included more than 10 patients. Duchowny et al. studied 13 patients with electrocorticographic recordings defining seizures starting from the temporal posterior (Duchowny et al. 1994b) base.^34^ They found a preferential spreading to the parietal and/or the frontal convexity. They describe a common semiology in the majority of cases, characterized by behavioral arrest, visual hallucination (2/10 cases), and a delayed appearance of motor signs (version, contralateral dystonia, clonic jerks, and automatisms). Mirandola et al.^5^ studied 14 patients with parahippocampal/inferior temporal (PIT) lesions compared to 36 with hippocampal sclerosis (HS). The seizure duration on scalp EEG was identical between the two groups (mean = 70 seconds) but the semiological features emerged more quickly in the inferior temporal group. The clinical manifestations differed between the two groups: motionless staring was more frequent in the HS group (81%, n = 20) compared to the basal temporal group (43%, n = 6, *p* < .05), whereas hypermotor bilateral automatisms were exclusively observed in the PIT group (43%, n = 6, *p* < .05). In the study by Hadidane et al.^7^ 24 patients with an epileptogenic zone located in the basal temporal region were investigated using SEEG, representing 8% of the SEEG cases. Patients had a mean age at SEEG of 28 years (range 9.5–53 years). A large proportion were lesional (83%), with the majority having malformations of cortical development. Some semiological features differed between left‐and right‐side seizures: aphasia (mainly naming difficulties in 71% of cases as an initial sign, but also jargonophasic production) occurred frequently in left‐side basal temporal seizures, while verbal automatisms were frequent in right‐side basal temporal seizures (54%). Toledano et al.^8^ studied the electroclinical features of seizures in 15 patients with temporopolar and basal temporal lesions to identify specific characteristics that distinguish them from seizures originating in other temporal regions. Among 172 patients with temporal lobe seizures, 15 (8.7%) had seizures caused by temporopolar or anterior basal temporal lesions, with 11 of these lesions located on the left side. Their main finding was that aphasia emerged as the most prominent feature during seizures in patients with left‐sided lesions. Additionally, in the study by Trebuchon et al.^40^ the early involvement of the posterior occipitotemporal sulcus was associated with jargon aphasia.

## 4 DISCUSSION

In this review, we sought to characterize the clinical semiology of seizures originating in the basal temporal region. Our literature search revealed that studies on these epilepsies often lack precise anatomical definitions, particularly when detailed invasive exploration is absent. Notably, only one study to date^7^ has accurately defined the epileptogenic zone and anatomical boundaries of these epilepsies using SEEG quantification. Diagnosing basal temporal seizures remains challenging due to frequent overlap with other temporal lobe epilepsy (TLE) subtypes. SEEG is crucial for differentiating these subtypes, as scalp EEG typically fails to detect activity confined to the basal temporal region. SEEG findings indicate that epileptogenicity is most frequently localized to the anterior fusiform gyrus, rhinal cortices, and parahippocampal cortex, often following an anterior‐to‐posterior gradient of seizure onset.^7^

Our semiological analysis highlights certain features that may help distinguish basal temporal seizures from other forms of TLE. Subjective semiology appears less rich, with infrequent emotional and sensory signs. However, experiential signs such as déjà vu or reminiscences occur in approximately 15% of cases, aligning with the anterior basal temporal cortex's (rhinal) known role in generating these phenomena.^41^, ^42^ Sensory symptoms are rare, likely reflecting the involvement of adjacent cortices, with visual symptoms being infrequently observed.

Objective semiology, on the other hand, may be more distinctive. Several studies emphasize the language and verbal components in these seizures, with aphasic features frequently emerging early in seizures originating from the dominant hemisphere.^4^, ^7^, ^8^, ^10^ These language disturbances commonly include naming difficulties, verbal production errors, or jargonophasia. The high prevalence of language alterations reflects the anterior basal temporal region's role as a transmodal hub for semantic processing and higher‐order cognitive functions.^22^, ^25^ Speech disturbances were particularly analyzed in a SEEG‐based publication of basal temporal seizures.^7^ This paper showed that naming deficits and anomia were significantly more prevalent in left basal temporal epilepsy (71% vs. 29%, *p* < .001). Automatic speech production (verbal automatisms, jargonaphasia) was significantly more common in right‐sided basal temporal epilepsy (54% vs. 11%, *p* = .001). Early naming difficulties are strongly associated with left EZ, while late comprehension deficits are more likely associated with posterior basal temporal involvement. Surgical intervention in basal temporal lobe epilepsy can significantly impact language functions, particularly when the resection involves language‐dominant regions. Studies have shown that 30%–50% of patients undergoing resection in the language‐dominant hemisphere experience notable postoperative language decline, such as word‐finding difficulties, which can profoundly affect daily life.^43^ The (BTLA) plays a crucial role in language processing. Resection of the basal temporal language area has been associated with specific and early naming declines.^24^ Although some patients may experience transient deficits, naming scores often remain lower than baseline even after recovery.^24^ However, while ictal semiology aids in localizing the EZ, its utility in predicting the safety and efficacy of surgical interventions concerning language outcomes is limited. Comprehensive presurgical evaluations, including functional mapping and neuroimaging, are essential to assess potential risks to language function and to guide surgical planning.^44^

Motor phenomena, including tonic, clonic, spasms, orofacial movements, or automatisms, are also observed. Hyperkinetic manifestations, reported in some series,^5^ suggest an overlap with the temporopolar region, known for its role in hyperkinetic seizure genesis.^45^ Additionally, rare semiological features, such as gelastic or mirth manifestations, have been documented and may point to the basal temporal cortex's involvement in laughter.^31^, ^32^ Regarding this feature, a recent study showed how the fusiform gyrus, together with the temporal pole and middle and inferior temporal gyrus are involved in the sense of humor.^46^

## 5 CONCLUSIONS

The aim of our study was to characterize seizures originating in the basal temporal region and to distinguish them from other subtypes of temporal lobe epilepsy. The data in the literature are fairly heterogeneous and do not allow us to identify a completely characteristic clinical picture. Certain semiological trends have been observed, but no statistically significant marker has been identified to distinguish basal temporal seizures from temporo‐lateral or temporo‐polar epilepsy. In addition, the overall level of evidence remains limited due to the heterogeneity of the studies, the limited sample sizes, and the absence of controlled comparisons.

There is moderate evidence that basal temporal seizures are associated with language impairments (aphasia, jargonaphasia), somatomotor or somatosensory facial manifestations, hyperkinetic manifestations, behavioral arrest, and emotional/affective symptoms such as ictal pleasant sensations. This association is strong at the group level, as multiple studies identified consistent patterns of seizure semiology linked to basal temporal involvement. However, at the individual patient level, variability exists due to differences in seizure onset zones, propagation patterns, and functional brain organization.

Future studies should aim to improve methodological rigor by using standardized criteria for assessing semiology and incorporating comparative analyses with other subtypes of temporal lobe epilepsy. In addition, statistical validation of the proposed clinical features will be essential to confirm that basal temporal epilepsy has truly distinct characteristics. Until such data are available, caution should be exercised in interpreting the clinical relevance of basal temporal seizure patterns.

## CONFLICT OF INTEREST STATEMENT

None of the authors has any conflict of interest to disclose.

**Test yourself**

1. Which anatomical structures are frequently implicated as epileptogenic zones in basal temporal seizures? (Select all that apply)
  - Fusiform gyrus
  - Parahippocampal gyrus
  - Amygdala
  - Rhinal cortex
  - Temporal pole
  Answers: ABD
2. Which semiological features are commonly associated with basal temporal seizures? (Select all that apply)
  - Aphasia/Jargonophasia
  - Déjà vu
  - Oro‐alimentary automatisms
  - Hyperkinetic behaviors
  - Epigastric sensations
  Answers: ABD
3. Which semiological symptoms are less commonly observed in basal temporal seizures? (Select all that apply)
  - Fear
  - Auditory hallucinations
  - Epigastric sensations
  - Somatosensory symptoms
  - Jargonophasia
  Answers: ABCD

## Data Availability

The data that support the findings of this study are available on request from the corresponding author. The data are not publicly available due to privacy or ethical restrictions.
